# Sensitization of Drug Resistant Cancer Cells: A Matter of Combination Therapy

**DOI:** 10.3390/cancers10120483

**Published:** 2018-12-04

**Authors:** Meghan Leary, Sarah Heerboth, Karolina Lapinska, Sibaji Sarkar

**Affiliations:** 1 Boston University School of Medicine, Boston, MA 02118, USA; meghanl@bu.edu; 2 Vanderbilt University School of Medicine, Nashville, TN 37240, USA; sarah.a.heerboth@vanderbilt.edu; 3 Grand Strand Medical Center, Myrtle Beach, SC 29572, USA; karolinaeva1@gmail.com; 4 Division of Biotechnology, Quincy College, Quincy, MA 02169, USA; 5 Division of Biology, MBC College, Wellesley, MA 02481, USA; 6 Division of Biology, RC College, Boston, MA 02120, USA

**Keywords:** cancer drug sensitization, drug resistance, combination therapy, stem cell, cancer progenitor cell, cancer therapy, chemotherapy, epigenetics, methylation, histone modification

## Abstract

Cancer drug resistance is an enormous problem. It is responsible for most relapses in cancer patients following apparent remission after successful therapy. Understanding cancer relapse requires an understanding of the processes underlying cancer drug resistance. This article discusses the causes of cancer drug resistance, the current combination therapies, and the problems with the combination therapies. The rational design of combination therapy is warranted to improve the efficacy. These processes must be addressed by finding ways to sensitize the drug-resistant cancers cells to chemotherapy, and to prevent formation of drug resistant cancer cells. It is also necessary to prevent the formation of cancer progenitor cells by epigenetic mechanisms, as cancer progenitor cells are insensitive to standard therapies. In this article, we emphasize the role for the rational development of combination therapy, including epigenetic drugs, in achieving these goals.

## 1. Introduction

### Drug Resistance in Cancer and Relapse

**Figure 1:**
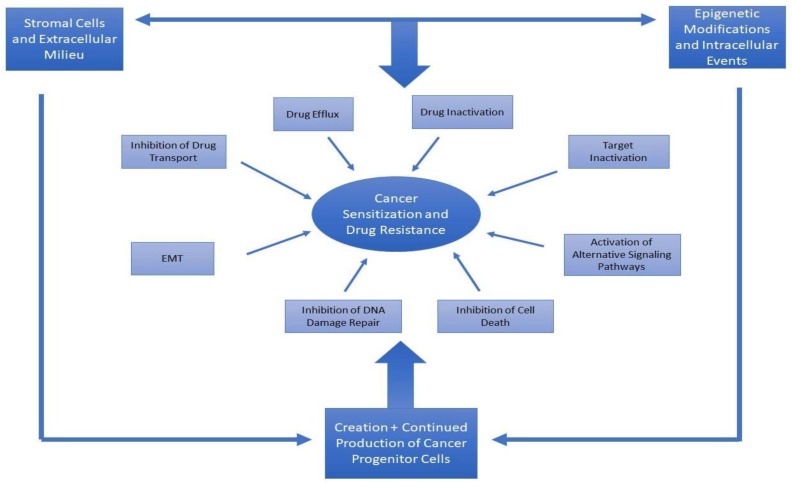
This figure demonstrates a model of possible causes of cancer drug resistance. All of these pathways could be regulated by extracellular signaling, epigenetic events, and intracellular events and signaling. A combination of all these elements produces cancer progenitor cells and sustains their production, even when cancer is apparently in remission.

Drug resistance poses a major problem to cancer therapy, as most chemotherapy will produce resistance after prolonged use. The causes behind the development of resistance are multi-fold and often include different mechanisms. Some of the mechanisms of resistance are shared between microbes and cancer cells, which suggests that drug resistance is an evolutionary process [1,2]. The causes of cancer drug resistance include: efflux, target alteration, enhanced and alternative metabolism, alteration of cell surface receptors, active stroma, drug degradation, escape from apoptosis and immune system evasion, DNA damage repair, epithelial mesenchymal transition, and epigenetic alterations regulating one or more of these processes (Figure 1) [2,3,4]. A recent study supported the notion that drug resistance in cancer is a multifaceted process. The main challenge in understanding all aspects of this complicated process is the scarcity of sampling data. The authors pointed out that the development of cancer resistance is a cumulative effect of all the targets and pathways that are affected by a particular drug treatment. When we consider how to develop a strategy to combat drug resistance, we need to consider the convergence of different aspects of resistance [4]. Two other important mechanisms implicated in cancer drug resistance are the continuous development of tumor cells from existing cancer stem/progenitor cells, and the survival of the cancer stem/progenitor cells after chemotherapy [2]. These two important points are often overlooked when cancer patients are treated, and when they become clinically cancer-free. It appears that if these two processes are not addressed, the cancer will relapse in most cases. As these processes are epigenetically regulated, we argue that using a combination of epigenetic drugs with other therapies will improve the outcome [5,6,7,8,9,10]. In this article, we will discuss the sensitization of drug resistant cancer cells and the rationale to develop such therapies.

## 2. Sensitization and Cancer Progression

### 2.1. Cancer Drug Resistance

The hallmarks of cancer describe the common pathways that are known to be involved in carcinogenesis [11,12]. These pathways include self-sufficiency and growth signaling, insensitivity to anti-growth signals, limitless reproductive potential, tissue invasion and metastasis, resistance to apoptosis, sustained angiogenesis, immune surveillance evasion, tumor-promoting inflammation, genome instability and mutation, and dysregulation of cellular energetics. Most of the chemotherapies currently in use inhibit at least one of these pathways, directly or indirectly, but cancer cells become insensitive to the drugs after prolonged use (Figure 1).

The development of cancer drug resistance poses two problems: it increases relapse rates and makes treatment of the resistant cancer virtually impossible. The concept of sensitization involves making the drug resistant cancer cells sensitive to the same or different drugs, in order to develop a successful therapeutic regimen. The first step of sensitization is to reverse the process of insensitivity. If this cannot be achieved, then the second step would be to inhibit an alternative pathway to facilitate the death of cancer cells using either the same drug or different drugs. This is warranted, due to the fact that we are running out of targets to develop new drugs [13].

The sensitization process increases the likelihood of survival among cancer patients who relapsed after cancer remission [2]. This concept has been proposed for several years, and it is rooted in the fact that numerous pathways are involved in carcinogenesis [5,6,7,8,9,10,12]. Some of these same pathways are involved in the development of drug resistance [2].

### 2.2. Oncogene Addiction

Conventional wisdom suggests that carcinogenesis is the result of multiple stepwise mutations [14]. Current studies have contradicted this paradigm and they propose that carcinogenesis (in terms of cancer progenitor cell formation) may be an epigenetic event that encompasses both histone modification and DNA CpG methylation [5,6,7,8,9,10]. The epigenetic hypothesis explains that mutations drive tumor progression once cancer progenitor cells have been formed. The initiation of this progression makes the cancer progenitor cells genetically unstable, favoring the accumulation of numerous somatic mutations. This has been observed in almost all cancer lineages [5,6,7,8,9,10,15]. Interestingly, only about 100 different mutations out of many can actually drive the progression of cancer. These are known as driver mutations. Recent bioinformatics research based on individual pathway analysis has produced several linkmaps, which can predict which combination of mutations will favor which type of cancer. The dependence of cancer on these mutations to maintain and grow is referred to as oncogene addiction [16]. An example of oncogene addiction is found in Myc overexpression in osteosarcoma. The inactivation of Myc caused tumor cells to reform normal bone [17,18]. It has been demonstrated that colon cancer progression requires sequential mutation of APC, K-Ras, and p53 [19,20]. A large-scale genome analysis of more than 3000 tumors from 12 different cancer lineages found that mutations in TP53, DNTM3A, and PIK3CA occurred early, contributing to the initiation of carcinogenesis, whereas mutations in K-Ras and N-Ras occurred later and were associated with cancer progression rather than initiation [21,22].

The past three decades have seen exponential growth in targeting mutations to treat diverse types of cancer. In one study, the authors found that by targeting particular oncogenes such as HER-2, BCR-ABL, and EGFR, they were able to cause cancer cells to revert to a noncancerous phenotype and arrest tumor growth [16]. A problem, however, arises when cancer cells rapidly develop resistance to the tyrosine kinase inhibitors and other small molecule inhibitors that are used to target particular pathways. Sellers et al. in 2011 demonstrated that cancer cells consistently express a pattern of resistance mechanisms to restore the activity of the oncogene that is maintaining their phenotype [23]. For example, lung adenocarcinoma with an EGFR mutation will develop resistance to EGFR inhibitors, either through additional EGFR mutations or through the de novo mutation of MEK. Both mutations will act to restore downstream activity of PIP3 [24,25]. Similarly, BRAF melanomas will overcome BRAF inhibition via the reactivation of the MEK–ERK cascade [23]. 

Therefore, it is necessary to target multiple pathways in order to combat drug resistance. Horizontal pathway combinations involve the targeting of two or more separate pathways at once. Vertical pathway combinations involve the use of both upstream and downstream inhibitors [23]. However, when designing new combinations of small molecule inhibitors, it is important to ensure that the pathways being targeted do not antagonize another pathway.

A mutation profile of breast cancer identified 40 mutation-driver genes in breast cancer. The authors found that a high level of intra-tumor heterogeneity is associated with worse prognosis [26]. A recent mathematical model analyzed the mutations in self-renewing tumor tissue and found that the number of mutations was positively correlated with the age of the patient at the time they were diagnosed. Their analysis suggested that half or more of somatic mutations in these tumors occurred before the initiation of neoplasia [27]. Furthermore, in a model of adult stem cells, researchers found that liver stem cells had a different mutation profile than stem cells from the colon and small intestine, although all groups of stem cells had a similar mutation rate. They found that the liver stem cells’ mutations were more similar to the mutation profiles seen in cancer, suggesting that intrinsic mutation properties in these stem cells are implicated in carcinogenesis [28]. These findings are consistent with an epigenetic model of carcinogenesis, as we propose that although these cells have accumulated a great number of mutations, they do not progress to the initiation of carcinogenesis until the epigenetic landscape within these cells favors the progression of cancer development [10,15,29].

## 3. Examples of Drug Resistance

### 3.1. Drug Inactivation

Many cancers have developed mechanisms to overcome chemotherapeutic agents, either by inactivation of the drug (for example the inactivation of platinum drugs by thiol glutathione, which activates the detoxification system of the cell) [30,31] or by the decreased conversion of the inactive substrate to the active form of the drug. A major mechanism of drug inactivation occurs through the cytochrome P450 (CYP) system. Class I of the P450 system consists of CYP1A1, CYP1A2, CYP2E1, and CYP3A4, and these enzymes are involved in the metabolism of drugs and procarcinogens. Class II consists of CYP2B6, CYP2C9, CYP2C19, and CYP2D6 and is also involved in drug metabolism. It is theorized that mutations in the CYP system may lead to the increased breakdown and excretion of chemotherapeutic drugs [32,33,34,35]. The GST superfamily is a group of enzymes that contributes to drug resistance through the detoxification of chemotherapeutic drugs [36,37]. The UGT superfamily is a group of enzymes involved in glucuronidation, and are the cell’s first line of defense from environmental carcinogens and toxins. Downregulation of a particular gene in this family, UGT1A1 is negatively regulated via DNA methylation, and is implicated in resistance to irinotecan (topoisomerase inhibitor) [32,38,39]. Conversely, failure of a cell to convert a drug to its active form may confer resistance to that drug. A prominent example of this mechanism of resistance is observed with the nucleoside drug cytarabine (AraC). This drug is activated by phosphorylation which converts AraC to AraC-triphosphate. Downregulation of the phosphorylation pathway leads to decreased activated AraC, and therefore AraC resistance [40,41]. The above mechanisms are frequently under epigenetic control, with DNA methylation-silencing genes that are necessary to activate drugs, or decreased methylation causing increased expression of proteins that inactivate drugs [38,42].

### 3.2. Epithelial–Mesenchymal Transition

The epithelial–mesenchymal transition (EMT) is the process by which cells de-differentiate into a mesenchymal phenotype. During EMT, tumor cells downregulate the expression of cell–cell adhesion molecules such as integrins, cadherins, and selectins. Increased expression of metalloproteases on the cell surface assists with breaking down the extracellular milieu, allowing the tumor cells to move away from their original location [2,8]. This process promotes metastasis, and it has been implicated in drug resistance [8,43,44]. Many of the markers that are upregulated in EMT are also strong promoters of cell survival. Transforming growth factor β (TGF-β) is a vital mediator of EMT [8] and it is also implicated in increased resistance to chemotherapy drugs [45]. In metastatic breast cancer, high levels of CD44 (marker of EMT associated with more aggressive tumor growth) and low levels of CD24 are linked to drug resistance [38,46,47]. HER-2-positive breast cancers that express high levels of the β1 integrin have higher rates of resistance to trastuzumab [48].

### 3.3. Drug Efflux

The MDR (multidrug resistance) protein is a membrane-bound protein that is overexpressed in many tumors at baseline, and its expression can be induced by the exposure of the cancer cells to chemotherapy. For example, lung cancer cells were found to have an increased expression of MDR1 following exposure to doxorubicin [49]. Other similarly functioning drug efflux pumps have been identified, including MRP1 and BCRP, and these are also associated with drug resistance in a variety of cancers. These proteins are able to clear a multitude of chemotherapeutics such as vinca alkaloids, anthracyclines, and taxanes, as well as small-molecule inhibitors, including imatinib and erlotinib, from the cell. CD44, which was noted before to be associated with EMT and increased metastasis, has been found to correlate with the level of MDR proteins [38,50,51].

### 3.4. Inhibition of Transport

Similar to how cancer cells are able to resist chemotherapy by the enhanced efflux of drugs, they have also developed mechanisms to decrease influx of the drug into the cell by active transport. For example, imatinib, a tyrosine kinase inhibitor used in chronic myeloid leukemia (CML), is transported into cells via the organic human cation transporter 1 (hOCT1) [52]. Increased expression of hOCT1 is associated with a better response to treatment with imatinib [53]. 

### 3.5. Target Alteration

Alteration of the drug target is a major mechanism of resistance to small molecule inhibitors, due to their specificity. For example, many cancers are driven by mutations in oncogenic kinases, such as the Raf family and EGFR. Drugs such as erlotinib were developed to specifically target constitutively-active EGFR; however, in many cancers, secondary mutations in EGFR rapidly develop to overcome the inhibition [25]. Another example is resistance to imatinib, which targets the BCR-ABL protein in CML. Mutations in the imatinib binding site inhibit the action of the drug while preserving the function of the fusion protein [38,54].

### 3.6. DNA Damage Repair

Many chemotherapeutic drugs act to damage DNA, triggering cell cycle arrest and stopping the overactive replication of cancer cells. For example, platinum drugs, alkylating drugs, and topoisomerase inhibitors all act to disrupt the DNA replication process. Increased expression of proteins within the nucleotide excision repair group is associated with resistance to the platinum-based chemotherapy cisplatin in ovarian cancer cell lines [55]. Upregulation of CD133 in glioblastoma cells has been found to contribute to resistance to radiation therapy, via the activation of DNA damage checkpoints and the increased repair of radiation-induced DNA damage [56]. Certain chemotherapies induce DNA damage through guanine-O6 alkylation. The O6-methylguanine DNA methyltransferase functions to repair the alkylated guanine molecule before mismatch mutation can occur. Overexpression of this protein is seen in many tumors, conferring resistance to alkylating chemotherapeutic agents [57].

### 3.7. Cell Death Inhibition

Resistance to cell death (i.e., resistance to apoptosis) is one of the hallmarks of cancer, making it a target for cancer therapy. Many cancer cells express high levels of anti-apoptotic proteins, allowing them to escape programed cell death. As reducing tumor growth is one of the main goals of chemotherapy, and induction of apoptosis is a major mechanism by which traditional chemotherapeutic drugs function. However, many cancers have developed mechanisms to bypass chemotherapy-induced apoptosis. For example, in small cell lung cancer, researchers have found that increased expression of protein tyrosine kinase is associated with resistance to induced apoptosis via integrin survival signaling mechanisms [38,58]. 

### 3.8. Tumor Heterogeneity 

One of the most challenging aspects of cancer treatment is tumor heterogeneity. Every tumor is composed of groups of different types of cells, in terms of mutations and epigenetic markers. But essentially, they arise from a small group of cancer progenitor cells that is not that diverse. Further mutations are acquired during tumor progression. For example, in samples of different breast cancers, more than 2000 mutations were observed, among which a limited number are considered to drive cancer progression [10,15]. Byler et al. have thoroughly reviewed these aspects, and suggest that as the apparent heterogeneity arises from a few cancer progenitor cells, and if we believe that cancer progenitor cell formation is an epigenetic event, then the introduction of epigenetic drugs could reduce the level of heterogeneity during treatment following cancer remission, as they will prevent the root cause, the formation of cancer progenitor cells [10,15]. 

## 4. Current Combination Therapies in Use

Once it was established that carcinogenesis and the maintenance of continuous cancer cell growth depends on several pathways, combination therapy was developed to achieve better outcomes. Traditional chemotherapeutic agents are frequently used in combination when treating leukemia and lymphoma. In diffuse large B-cell lymphoma, expression of Bcl-2 is associated with poor prognosis and a decreased efficacy of chemotherapy. Clinical trials found that the addition of rituximab to the standard therapy (CHOP—cyclophosphamide, doxorubicin, vincristine, and prednisone) had higher response rates and 2-year overall survival and event-free survival rates [59]. Solid tumors are also treated with combinations of often several drugs at once. For recurrent or persistent ovarian cancers or high-grade stromal tumors, bevacizumab (VEG-F inhibitor), PARP inhibitors, and/or hormone therapies are combined with a chemotherapy regimen [60]. Ovarian cancers have a high recurrence rate, with 80% of patients with advanced ovarian cancer experiencing recurrence within a few months of surgery and chemotherapy, and these cancers often develop resistance to traditional chemotherapy [61,62]. 

Liver cancer is often resistant to most chemotherapeutic drugs, and no combination is superior to single agents. Most combinations in use include Cisplatin and/or doxorubicin. Many of the combinations in use to treat liver cancer are associated with significant toxicity [63]. Current combinations in use for breast cancer include Doxorubicin/epirubicin ± taxanes ± 5-FU ± cyclophosphamide ± carboplatin (+trastuzumab if HER-2 positive). A stage III clinical trial compared the combination of gemcitabine + paclitaxel vs paclitaxel alone in metastatic breast cancer. Multidrug therapy was found to have a higher median overall survival than monotherapy, was associated with a higher survival probability at 12, 18, 24, and 30 months, and had a longer time to disease progression [64].

Noscapine (Nos, a benzylisoquinoline alkaloid) has been used in clinical trials in low concentrations to sensitize triple negative breast cancer to docetaxel, a traditional chemotherapeutic drug. They found that Nos inhibited the proliferation of resistant and non-resistant breast cancer cells. The combination of Nos and docetaxel showed significant reduction in tumor volume compared to either drug alone. The combination was found to downregulate anti-apoptotic proteins, as well as multidrug resistance proteins [65]. Paclitaxel is a first-line therapy for breast, lung, and ovarian cancers. However, successful therapy is frequently hindered by the development of resistance to paclitaxel through elevated expression of prohibitin1 (PHB1) and GSTπ. Small interfering RNAs (siRNAs) have been successfully used to silence PHB1 and GSTπ to partially increase sensitivity to paclitaxel via the activation of intrinsic apoptotic pathways [66].

## 5. Problems with Current Combination Therapies 

The concept of combination therapy has been proposed for the past 20 years, and is becoming accepted and integrated into common clinical practice. Examples of some epigenetic combination therapies currently in use are described in a later section. Researchers have found that carcinogenesis is the outcome of aberrant regulation across diverse pathways, as described above [4]. While this makes developing treatments for cancer difficult, it is possible to take advantage of this to develop future therapies. When a particular therapy shuts down one pathway, a cancer may develop resistance through the activation of alternative pathways. The advantage comes in that this process can be exploited for treatment with drugs that target these alternative pathways. The alternate pathways could be utilized either to sensitize or to kill, or both. This rationale led to the idea of combination therapy. As much as double coverage with multiple antibiotics are sometimes used to treat resistant bacteria such as *Pseudomonas*, multiple chemotherapies can be used to treat resistant cancers.

**Figure 2:**
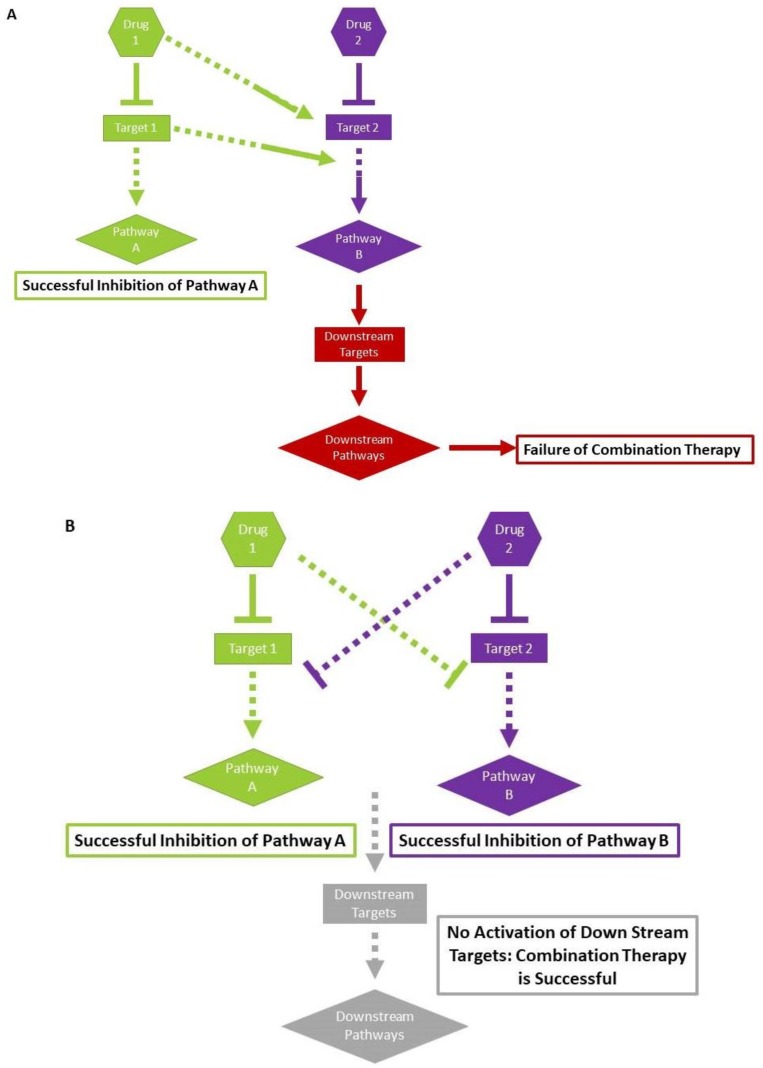

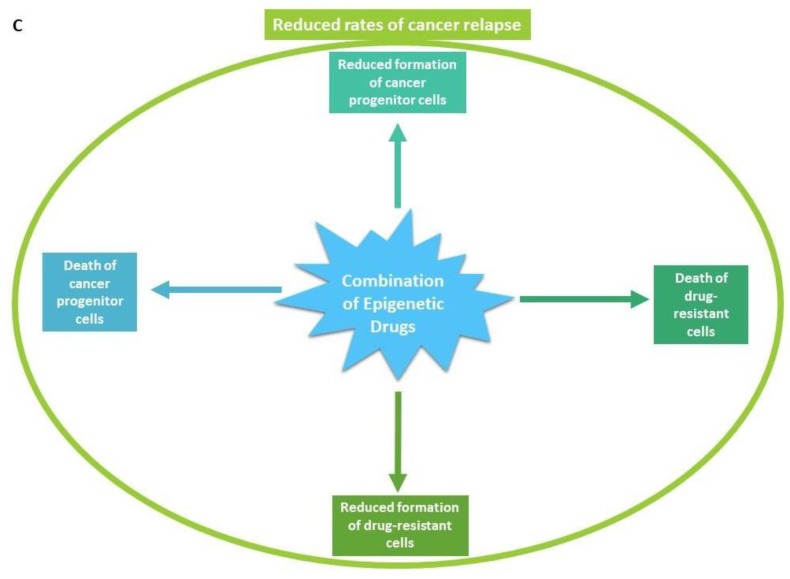
(**A**) Figure 2A depicts a model of antagonistic combination therapy. Drug A, a metabolic product of Drug A, or one of the downstream effectors blocks the effect of Drug B, or the downstream pathway of Drug B, inhibits the effect of combination therapy. (**B**) Figure 2B depicts a model of synergistic combination therapy. Drug A, a metabolic product of Drug A, or one of the downstream effectors, enhances the effect of Drug B or the downstream pathway of Drug B, and vice versa, enhancing the effect of combination therapy. (**C**) Figure 2C depicts a model of the positive effects of a combination therapy including epigenetic drugs. This combination kills the cancer progenitor cell, sensitizes cancer drug-resistant cells, inhibits the production of drug-resistant cancer cells, and inhibits the production of cancer progenitor cells.

However, an often-overlooked point is that sometimes pathways and targets are antagonistic. For example, in many cell types, it was observed that if ERK is inhibited, Akt activity is increased (Figure 2A) [67,68]. We need to remember that both kinases play significant roles in carcinogenesis [67]. These types of contradictions often lead to a failure in the desired effect of the combination therapy. Even in dysregulated cancer cells, there exists a delicate balance between signaling pathways, and we must strive to predict how one therapy targeting one pathway may lead to unexpected changes in other pathways.

There are many issues that limit the utility of target specific small molecule inhibitors. For one, they must match the genetic profile of the target cancer. For example, the tyrosine kinase inhibitor imatinib is only effective in cells with the specific BCR-ABL fusion protein. As cancers are heterogeneous populations of cells, this makes it difficult to specifically profile and match inhibitors to the target protein, but it also means that certain cells within the population may be carrying genes that confer inherent resistance to certain inhibitors [69]. Target-specific inhibitors tend to lend themselves to the rapid development of resistance. Point mutations in target proteins can alter binding sites and render these drugs ineffective. In addition, the inhibition of one target pathway means that alternative oncogenic pathways can develop in response to the inhibitors. This mechanism of resistance is linked to the concept of oncogene addiction, as described above. The overexpression of a certain pathway within a cancer cell does not necessarily mean that that pathway is the driver of carcinogenesis, and similarly, inhibiting this pathway does not mean a reversal of tumorigenesis. Also, pathways that are not previously identified as driver mutations may still be supporting cancer growth [16,23,38,70]. 

A recent study described the complication of pathway adaptation. An inhibitor of the BRAF V600E mutant works for a few hours in melanoma, colorectal, and thyroid cancer. This decreases the dependence of the tumor on this mutation for that time. However, the tumor may continue to use alternative pathways while the BRAF pathway is inhibited. If this drug is used for a prolonged amount of time, the tumor develops resistance to the original drug and keeps the alternative pathway active. The authors pointed out that the combination of MAP kinase inhibitors with this inhibitor showed better outcomes, as the MAP kinase inhibitor acted downstream of alternative pathway activation (Figure 2A) [71,72,73]. Similarly, suppression of overactive PI3 kinase in ER+ breast cancer leads to the strengthening of ER receptor signaling [74]. This may be advantageous in combination therapy design, as the increased expression of ER is a target for specific therapies such as tamoxifen. 

A 2017 article pointed out that the recent developments in immunotherapy, which seem promising in cancer treatment, may also contribute to drug resistance. Tumor cells generally learn how to adapt to the immunologic attack. Current immunotherapy is designed to target specific tumor cells with the immune system, so that they cannot evade it. However, the prolonged use of antibodies raises a possibility that the tumor cells will acquire resistance to them. This risk increases with the development of heterogeneity within tumors. Therefore, combination therapy, including immunotherapy, is already being tested to overcome this problem [75]. 

## 6. Rationally Designed Combination Therapy

Rational combination therapy takes some important points into consideration. The first step is considering the type of cancer or tumor. If it is a tumor, how heterogeneous is it? What is known about the aberrant signaling and mutation profiles in this type of tumor? Are there identified driver mutations? These aspects are already considered when choosing a single chemotherapeutic agent. Rational combination therapy will consider which pathways and targets will work together and will increase the efficacy of the process. It may be possible to use a combination of drugs in classically suboptimal doses if they work together to have a synergistic or enhanced effect (Figure 2B). This is a huge advantage, as lower doses of chemotherapy lead to a decreased risk of side effects.

The delivery system and the pharmacokinetics of individual drugs also affect the process. The process of synergy sometimes relies on the fact that one drug will induce the effect of another drug, so that they cannot be administered simultaneously. When rationally designing, all of these variables need to be considered. These variables open a new area of study in bioinformatics that will combine the targets/pathways with the effect of individual drugs and their effect over time for whether they will produce better or worse outcomes. Essentially, such bioinformatics modeling analysis is already in place when small molecules or biologics are designed as potential chemotherapeutic agents. 

A recent article described the systems biology strategy for modeling the cellular and molecular regulatory circuits of disease states, steady states, and those states in between. This modeling helps to predict how many pathways need to be targeted to overcome resistance and achieve a good outcome. The author describes a rational model of choosing drugs based on physiology-based hypotheses. For example, a blockade of CTLA-4 by monoclonal antibodies results in compensatory activation of PD-1 receptor and its ligands, leading to resistance [76]. Simultaneous targeting of CTLA-4 and PD-1 overcomes this resistance. In the context of the development of a bioinformatics program, Mayer et al. recently described a delivery technology platform that determines the administration of chemo at suboptimal doses in combination, considering both their mode of action and pharmacokinetics. They provided an example where the alteration in drug ratio may provide either synergistic or antagonistic effects. Treatment with irinotecan and cisplatin at a 1:1 ratio was found to be highly antagonistic. However, at a 4:1 ratio, the combination was synergistic. In contrast, irinotecan and floxuridine at 1:1 ratio was synergistic, but at a 10:1 ratio it was antagonistic [77]. 

While creating such combinations is a good start, current treatment paradigms fail to consider the epigenetic aspects of combination therapy and how cancer stem/progenitor cells must be targeted.

## 7. Epigenetics and Combination Therapy

Our laboratory has hypothesized that carcinogenesis is initiated by the formation of cancer progenitor cells, which is an epigenetic event [2,5,7,8,10,15]. The importance of cancer progenitor cells has been recently realized because of their inherent resistance to chemotherapy and radiotherapy [78]. These observations led researchers to study the presence and organization of these types of cells in cancer [79]. One of the recent observations was that a subpopulation of basal-like human mammary epithelial cells could spontaneously de-differentiate into stem cell-like cells [79,80]. Like this, a subpopulation of non-stem like cells can be altered to cancer stem/progenitor-like cells. The understanding of this process has a huge potential to treat cancer patients. The authors suggest that as these types of cells de-differentiate, targeting these types of cells may not have long-term effects [80]. We hypothesize that these types of differentiation processes are mediated by epigenetic changes. If we stop these epigenetic changes by using epigenetic drugs in a combination therapy, this will neither allow the formation of cancer stem/progenitor cells, nor any subsequent de-differentiation. 

Another study supports this notion by showing that the presence of cancer stem cells poses a problem for a better outcome after radiotherapy for head and neck squamous cell carcinoma. The authors suggest that more study is needed in order to detect biomarkers for these types of stem cells for better treatment [78]. Again, this is an example that suggests that a combination with epigenetic drugs may result in a better outcome, as epigenetic dugs may stop the formation of cancer stem/progenitor cells.

Apparent remission and further relapse of leukemia suggests that the inclusion of epigenetic drugs in a combination therapy presents a better outcome. Acute lymphocytic leukemia (often associated with a mutation in *DNMT3A*) usually relapses after remission [81]. A separate study found that higher methylation levels in the cells of similar leukemia patients were maintained indefinitely, even after remission [82]. We hypothesize that the presence of high methylation levels continues to help generate cancer progenitor cells, causing relapses [5,7,8,15,29].

We postulated previously that formation of cancer progenitor cells is an epigenetic event. This process occurs by the alteration of an epigenetic switch, in terms of histone modifications and DNA methylation [29]. DNA methylation can regulate gene expression both positively and negatively. An example of negative regulation is the methylation of upstream promoter regions, which blocks the binding of RNA polymerase II (Pol II) and thus inhibits the transcription of principally tumor suppressor genes [7,9]. In a systems biology study, we observed that DNMT1 is allosterically activated at the site of methylation of genes silenced in leukemia [83].

Positive regulation is a process by which methylation detaches H3K27me3 from DNA and relieves the inhibitory effect, thus favoring the expression of growth-promoting genes [5]. This phenomenon mainly occurs in the enhancer region of the gene. A recent study has shown that there is significantly lower methylation in the CpG sites in ER+ breast cancer cells compared to ER− cells in the genes ERa, GATA3, and FOXA1. In addition, the authors observed that methylation patterns at CpG sites also corresponded to the level of lymphocytic infiltration into the breast tumor tissue [84]. This suggests that enhanced expression of growth-promoting genes is favored by methylation, which is also affected by the presence of lymphocytes within the tumor stroma [85].

### Advantages of Epigenetic Combination Therapy

We first proposed the idea of combination therapy of cancer where one drug would be epigenetic and other drug(s) would be other types of target-specific or non-specific chemotherapies, according to the design that we described above. This combination is superior, as it sensitizes cancer cells to other types of drugs, and it has the potential to kill existing drug-resistant cancer cells, to stop the formation of new cancer drug resistant cells, to kill existing cancer stem/progenitor cells, and to stop the formation of new cancer progenitor cells (Figure 2C). As epigenetic mechanisms are involved in the epithelial mesenchymal transition [8], combination therapy, including epigenetic drugs, could be more effective in reducing metastasis.

In 2008, Frew et al. showed that breast tumors shrunk in mice when histone deacetylase inhibitors (HDACi) were combined with tumor necrosis factor-related apoptosis-inducing ligands (TRAIL) [86]. A 2014 study showed that the combination of epigenetic drugs with other drugs reduced the relapse rate in lung cancer patients [87]. HDACi, in combination with the telomere inhibitor GT-oligo, was more efficient in inhibiting ovarian cancer cells [88,89]. We have previously shown that HDACi, in combination with other drugs, enhanced the effects of the therapy. In combination with the calpain-protease inhibitor calpeptin, HDACi produced enhanced growth inhibition, cell cycle arrest, and the induction of apoptosis in breast and ovarian cancer cells [90,91]. Epigenetic drugs have been used to enhance the expression of antigens in ovarian cancer through the use of demethylating agents and interferons [92].

In diffuse intrinsic pontine glioma with H3K27M mutation, researchers found that the use of bromodomains and extra-terminal motif (BET) inhibitors in combination with EZH2 inhibitors showed better growth inhibiting effects than either inhibitor used individually [93]. Though combinations of multiple epigenetic drugs were initially successful, the effects were not sustainable. One of the possible reasons was that mutations in oncogenes and tumor suppressor genes and the activation/deactivation process of different pathways helped in the progression and maintenance of cancer cells. Thus, we hypothesize that even greater effects may be observed when epigenetic drugs are combined with traditional chemotherapeutics.

Some epigenetic drugs have already been successfully used in combination with more traditional chemotherapies. A 2017 study found that bromodomain inhibitors in combination with PARP inhibitors in epithelial ovarian cancer had a synergistic effect, reduced tumor burden, and extended survival. The authors believe that some of this effect was due to the epigenetic therapy activating type I interferon signaling [94]. In acute myeloid leukemia (AML), patients who were treated with valproic acid therapy (which has HDACi activity) followed by all-trans-retinoic-acid had better outcomes than patients who initially received the two drugs in combination. It is thought that the HDACi activity was necessary to re-express the repressed retinoic-acid signaling pathway [95,96]. A more recent clinical trial in older patients with AML found no difference in event-free and overall survival when comparing standard intensive therapies compared to therapies with the addition of valproic acid, but did find higher relapse-free-survival in the valproic acid group. The researchers found that patients with a mutation in NPM1 may have a higher response to valproic acid therapy [97].

**Figure 3:**
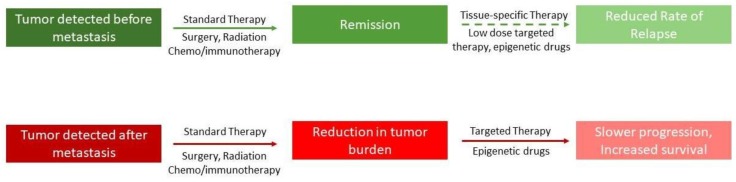
Figure 3 shows a model of how combination therapy, including epigenetic drugs, possibly affects the metastatic state of a tumor. The top panel shows that if the tumor is detected early, and if patients are given low doses of epigenetic drugs after apparent remission, it will reduce the chance of relapse. The lower panel shows that even if the tumor has metastasized, the likelihood of survival is increased if epigenetic drugs are introduced in combination.

Recent studies from different laboratories have also shown that the use of epigenetic drugs is capable of sensitizing drug-resistant cells to the same drugs, as well as to different drugs. In 2014, Cacan et al. showed that RGS 10 gene expression is epigenetically regulated, and that it causes platinum drug resistance in ovarian cancer cells [98]. Treatment of these drug-resistant cells with HDACi and DNMT1 inhibitors restored the sensitivity of these drug-resistant ovarian cancer cells to platinum drugs [99]. Similarly, in 2016, studies have shown that treatment of drug-resistant breast cancer with epigenetic drugs (HDACi and DMT1 inhibitors) blocked the tumorigenicity of the breast cancer stem cells [100]. The list of studies is increasing, which shows that not only a combination with epigenetic drugs enhances the efficacy of the combination in treating diverse types of cancers and tumors, but they can also make the cancer drug-resistant cells sensitive (Figure 3).

The epigenetic mechanism, which we called the “Epigenetic Switch,” involves both histone modifications and DNA methylation [5,10,15,29]. It is reasonable to believe that if epigenetic alterations lead to the development of cancer progenitor cells, then the inhibition of this process will stop the formation of cancer progenitor cells. We must consider another factor; many studies have shown that cancer progenitor cells are drug resistant, as well as resistant to radiotherapy [78]. If the cancer progenitor cells are formed by an epigenetic mechanism, then epigenetic therapy should also render these cells sensitized to other drugs. The combination of standard therapies, including target-specific drugs, with epigenetic drugs thus raises a possibility that the outcome of such treatments will be better than previously achieved (Figure 3).

The use of histone deacetylase inhibitors (HDACi) raises the issue of immunosuppression. However, recent studies reassure against this possibility. Valproic acid has been shown to trigger the differentiation of carcinoma cells, an important aspect that is reversed during carcinogenesis and that drives metastasis [8,101]. It is interesting to note that HDACi also reduces DNA methylation through the downregulation of DNMT1 [102]. Another study has shown that the use of HDACi at higher doses decreases the number of T-regulatory cells and natural killer cells in pleural mesothelioma. However, HDACi induces the expression of PD-L1 in these cells, increasing the likelihood that a combination therapy with anti-PD-L1 antibodies could be effective [103]. Briddle et al. have demonstrated that HDACi in combination therapy could be beneficial, as the therapy induces a T-cell response against tumor cells [104]. In a recent review, Kroesen et al. thoroughly discussed the pros and cons of HDACi effects on the immune system [105]. HDACis have both suppressive and activating effects on different immunogenic cells and cytokines, and studies have shown that HDACi in combination with immunotherapy produced synergistic effects, as they induced the expression of specific antigens on tumor cells, enhancing the effects of the immunotherapy. Further individual studies are necessary to determine the degree of epigenetic alterations in a particular patient, to tailor the therapy to the patient’s specific epigenetic profile. One option will be to combine lower doses of both HDACi and DNA methylation inhibitors with other standard therapies

Measurement of the cytosine methylation level is a non-invasive diagnostic tool that has been under investigation for the past several years. Researchers have used both circulating tumor cells and tissue samples, and determined the total methylation level in those tumor cells. In many types of cancers, higher methylation levels are an early indication of cancer development. It has been argued that the assessment of the methylation level of a given tumor could be used to determine a patient’s prognosis. We have proposed that since methylation is an epigenetic marker that is associated with the early development of cancer progenitor cells and drug-resistant cancer cells, then determination of the methylation level and location, whether upstream or within gene bodies, can be used to more specifically determine the utility of epigenetic drugs and their doses in combination therapy. In addition, following other treatments, the methylation level can be monitored to indicate how epigenetic drugs should be given as maintenance therapy.

Personalized medicine will need to look into both DNA methylation and histone modification in a cancer patient, to determine the type and dose of epigenetic drugs to be used in combination with other types of chemotherapy. A recent study in ovarian cancer showed that DNA methylation and histone modification differentially regulate gene expression in ovarian cancer cells, and are important biomarkers. The authors have shown that treatment with the DNA methylation inhibitor 5-Aza-dc and the histone deacetylase inhibitor TSA restricted cell cycling, decreased genetic instability, and re-expressed the tumor suppressor genes PTGIS [106]. Higher levels of methylation in leukemia patients have been investigated as a biomarker [82]. A systematic approach to determine epigenetic biomarkers in cancers is thoroughly discussed by Bock [107]. We postulated previously that a particular combination of DNA methylation and histone modification should be called the Epigenetic Switch, the alteration of which leads to the development of cancer progenitor cells [29].

## 8. Future Prospects

The successful management of cancer depends on two critical points. One is therapy. The other is the prevention of relapse. The past three decades have shown much improvement in therapy and in some types of cancer, and longevity has increased significantly. The most important problem that persists is the issue of relapse, which still occurs frequently. In this article, we propose points that are relevant to reducing the rate of cancer relapse, as well as the development of improved combination therapies. Extensive studies have been performed to identify not only signaling mechanisms, but also how different pathways interact with each other. Inhibitors of almost all known targets are either in use or in the development process, but it is frustrating that almost all of them generate resistance. This article emphasizes that a combination therapy that includes an epigenetic drug could be more useful in combating cancer cells and at the same time significantly reduce relapse. The inhibition of relapse will depend on which stage of tumor development the combination therapy is introduced. It is also important to understand that following standard therapy and during remission, epigenetic drugs should be introduced in suboptimal doses as a part of maintenance chemotherapy.

As discussed in the article, the introduction of each item in a combination therapy will depend on the pathways that they inhibit. An understanding of the DNA methylation and histone modification pattern will determine which epigenetic drugs should be introduced, when, and in what doses. Maintenance of epigenetic drug therapy will possibly prevent further cancer progenitor cell growth after remission. However, we must remember one cautionary measure in this endeavor. Epigenetic status throughout our life is in a flux and it alters as we age. Every cell type keeps its gene expression profile in check by these epigenetic mechanisms, in which genes are not altered, but differentially expressed. To artificially alter such a complex system could produce unwanted adverse effects. This is analogous to the use of 81 mg aspirin for cardiac patients; the low dose is able to decrease disease progression, and it also minimizes unwanted adverse effects. We may also learn from an apparent heterogeneity in our biological system. The entropy is high, but we perform extremely regulated processes within the chaotic environment.

The challenge is to find the window where both HDACi and DNA methylation inhibitors could be used in lower doses in combination with standard chemotherapies. The purpose of using epigenetic drugs will be to enhance the effects of other therapies, to help kill drug resistant cells by making them susceptible, prevent further production of cancer progenitor cells, and to reduce relapse. Further studies will reveal which type of epigenetic drugs will produce the best results in combination therapy, which is expected to differ from patient to patient, by the type of cancer, and by the dose and timing of the treatment when they are introduced. Nowadays, as we talk about personalized medicine, the introduction of epigenetic drugs provides a promise of better outcomes in cancer treatment and management.
